# MCT8 Deficiency in Females

**DOI:** 10.1210/clinem/dgaf311

**Published:** 2025-05-27

**Authors:** Stefan Groeneweg, Ferdy S van Geest, Floor van der Most, Lucia Abela, Paolo Alfieri, Andrew J Bauer, Enrico Bertini, Marco Cappa, Nurullah Çelik, Irenaeus F M de Coo, Anna Dolcetta-Capuzzo, Ilja Dubinski, Jorge L Granadillo, Lies H Hoefsloot, Vera M Kalscheuer, Marieke M van der Knoop, Heiko Krude, Kyle P McNerney, Laura Paone, Robin P Peeters, Catherine Peters, Markus Schuelke, Ulrich Schweizer, Jennifer E Sprague, A S Paul van Trotsenburg, Nina-Maria Wilpert, Ginevra Zanni, Laura J C M van Zutven, W Edward Visser

**Affiliations:** Academic Center for Thyroid Diseases, Department of Internal Medicine, Erasmus Medical Center, Erasmus University Rotterdam, 3015 GD Rotterdam, the Netherlands; Academic Center for Thyroid Diseases, Department of Internal Medicine, Erasmus Medical Center, Erasmus University Rotterdam, 3015 GD Rotterdam, the Netherlands; Academic Center for Thyroid Diseases, Department of Internal Medicine, Erasmus Medical Center, Erasmus University Rotterdam, 3015 GD Rotterdam, the Netherlands; Department of Paediatric Neurology, Universitäts–Kinderspital Zürich, 8008 Zurich, Switzerland; Unit of Child Neuropsychiatry, Department of Neurosciences, Bambino Gesù Children's Hospital, IRCCS, 00165 Rome, Italy; The Thyroid Center, Division of Endocrinology and Diabetes, The Children's Hospital of Philadelphia, Philadelphia, PA 19104, USA; Department of Paediatrics, The University of Pennsylvania, Philadelphia, PA 19104, USA; Unit of Neuromuscular and Neurodegenerative Disorders, Department of Neurosciences, Bambino Gesù Children's Hospital, IRCCS, 00165 Rome, Italy; Unit of Neuromuscular and Neurodegenerative Disorders, Department of Neurosciences, Bambino Gesù Children's Hospital, IRCCS, 00165 Rome, Italy; Division of Paediatric Endocrinology, Department of Child Health and Diseases, Sivas Cumhuriyet University Faculty of Medicine, 58140 Sivas, Turkey; Department of Paediatric Neurology, Erasmus Medical Center, Erasmus University Rotterdam, 3015 GD Rotterdam, the Netherlands; Mental Health and Neuroscience Research Institute, Department Translational Genomics, Maastricht University Medical Centre, 6229 ET Maastricht, the Netherlands; Academic Center for Thyroid Diseases, Department of Internal Medicine, Erasmus Medical Center, Erasmus University Rotterdam, 3015 GD Rotterdam, the Netherlands; Department of Endocrinology and Internal Medicine, San Raffaele Scientific Institute, 20132 Milan, Italy; Centre for Paediatric Endocrinology Zurich (PEZZ), 8006 Zurich, Switzerland; Division of Paediatric Endocrinology and Diabetology, Dr. von Hauner Children's Hospital, University Hospital (LMU), 80337 Munich, Germany; Department of Paediatrics, Division of Genetics and Genomic Medicine, Washington University School of Medicine, St Louis, MO 63110, USA; Department of Clinical Genetics, Erasmus Medical Center, Erasmus University Rotterdam, 3015 GD Rotterdam, the Netherlands; Research Group Development and Disease, Max Planck Institute for Molecular Genetics, 14195 Berlin, Germany; Department of Paediatric Neurology, Erasmus Medical Center, Erasmus University Rotterdam, 3015 GD Rotterdam, the Netherlands; Institut für Experimentelle Pädiatrische Endokrinologie, Charité–Universitätsmedizin Berlin, 10117 Berlin, Germany; Department of Paediatrics, Division of Endocrinology, Diabetes, and Metabolism, Washington University School of Medicine, St Louis, MO 63110, USA; Unit of Neuromuscular and Neurodegenerative Disorders, Department of Neurosciences, Bambino Gesù Children's Hospital, IRCCS, 00165 Rome, Italy; Academic Center for Thyroid Diseases, Department of Internal Medicine, Erasmus Medical Center, Erasmus University Rotterdam, 3015 GD Rotterdam, the Netherlands; Department of Paediatric Endocrinology, Great Ormond Street Hospital for Children, London WC1N 3JH, UK; Department or Neuropediatrics, Charité–Universitätsmedizin Berlin, 10117 Berlin, Germany; Institut für Biochemie und Molekularbiologie, Rheinische Friedrich-Wilhelms-Universität Bonn, D-53115 Bonn, Germany; Department of Paediatrics, Division of Endocrinology, Diabetes, and Metabolism, Washington University School of Medicine, St Louis, MO 63110, USA; Department of Paediatric Endocrinology, Emma Children's Hospital, Amsterdam UMC, University of Amsterdam, 1105 AZ Amsterdam, the Netherlands; Amsterdam Reproduction and Development Research Institute, 1105 AZ Amsterdam, the Netherlands; Department or Neuropediatrics, Charité–Universitätsmedizin Berlin, 10117 Berlin, Germany; BIH Biomedical Innovation Academy, BIH Charité Junior Clinician Scientist Program, Berlin Institute of Health at Charité–Universitätsmedizin Berlin, 10117 Berlin, Germany; Unit of Neuromuscular and Neurodegenerative Disorders, Department of Neurosciences, Bambino Gesù Children's Hospital, IRCCS, 00165 Rome, Italy; Department of Clinical Genetics, Erasmus Medical Center, Erasmus University Rotterdam, 3015 GD Rotterdam, the Netherlands; Academic Center for Thyroid Diseases, Department of Internal Medicine, Erasmus Medical Center, Erasmus University Rotterdam, 3015 GD Rotterdam, the Netherlands

**Keywords:** monocarboxylate transporter 8, MCT8, thyroid hormone, neurocognitive impairment, thyroid hormone transport, skewed X-chromosome inactivation

## Abstract

**Context:**

Monocarboxylate transporter (MCT) 8 facilitates thyroid hormone (TH) transport across the blood-brain barrier. Pathogenic variants in *SLC16A2* cause MCT8 deficiency (Allan-Herndon-Dudley syndrome), characterized by intellectual and motor disability and abnormal thyroid function tests. MCT8 deficiency typically affects males due to its X-linked inheritance.

**Objective:**

Here, we report 8 female patients with heterozygous pathogenic variants in *SLC16A2* who presented with variable neurocognitive impairment, behavioral problems, and TH function abnormalities.

**Methods:**

We performed X-chromosome inactivation studies in female patients in whom heterozygous pathogenic variants in *SLC16A2* were identified. The effect of *SLC16A2* variants on TH transport was assessed in transfected cells and patient-derived fibroblasts.

**Results:**

In all patients (mean age 8.6 years; range, 2.3-25 years) routine care genetic analyses identified heterozygous variants in *SLC16A2* (p.(R445C), p.(N193I), p.(G276R), t(X;20), resulting in a breakpoint in intron 1, t(X;19), resulting in a breakpoint in *SLC16A2*, p.(I562Sfs566*), p.(G221R)). All missense variants showed substantially reduced MCT8-mediated TH uptake in transiently transfected cells. X-chromosome inactivation studies in patient cells showed skewed X-inactivation in all 7 evaluated individuals. In 5 out of 7 evaluated cases, MCT8-mediated 3,5,3′-triiodothyronine (T3) uptake in patient-derived fibroblasts was impaired to a similar degree as in fibroblasts derived from male patients with MCT8 deficiency.

**Conclusion:**

Female patients with heterozygous pathogenic variants in *SLC16A2* and skewed X-chromosome inactivation may present with variable neuro(psycho)logical, behavioral, and thyroid function test abnormalities. Female patients presenting with neurocognitive impairment and abnormal TH function tests (low free thyroxine and/or high total T3 concentrations) should be tested for genetic variants in *SLC16A2*.

Thyroid hormone (TH) is essential for growth and development of virtually all tissues including the brain (1). Intracellular bioavailability of TH is governed by plasma membrane transporters (2). Monocarboxylate transporter (MCT) 8 is crucial for TH transport across the blood-brain barrier and into neural cells in the human brain (3-5). Pathogenic variants in *SLC16A2* (the gene encoding MCT8) cause MCT8 deficiency (Allan-Herndon-Dudley syndrome, AHDS) (6, 7). The hypothyroid state in the brain causes severe intellectual and motor disability, and failure to achieve early developmental milestones (8, 9). MCT8 deficiency has an estimated prevalence of 1:70 000 males (10). Most patients have severe hypotonia, hypokinesia, dystonia and spasticity, and do not develop speech (11). The typical endocrine fingerprint comprises high serum (free) 3,5,3′-triiodothyronine (T3) concentrations, low or low-normal (free) thyroxine (T4) concentrations, and high-normal thyrotropin (TSH) concentrations. Tissues that rely on TH transporters other than MCT8 are exposed to the high serum T3 concentrations, resulting in systemic thyrotoxic features, including tachycardia and failure to thrive (8).

Since *SLC16A2* is located on the X-chromosome (Chr.Xq13.2), pathogenic variants therein are known to typically affect males. Most female carriers do not present with clinically significant phenotypic abnormalities, since they have a second, unaffected, copy of the gene, resulting in the presence of a functional MCT8 in half of the cells (12, 13). However, skewing of the X-chromosome inactivation process in females with *SLC16A2* variants may lead to the majority of cells expressing mutant MCT8, resulting in a variable phenotype resembling MCT8 deficiency in males. This mechanism may also explain mild biochemical abnormalities and variable degrees of cognitive dysfunction reported in some female carriers (7, 14-16), while there is usually no skewed X-inactivation found in asymptomatic carriers (13). The potential of skewed X-chromosome inactivation leading to a phenotype resembling male MCT8 deficiency has been previously reported in 2 female patients, each harboring a different X-autosome translocation, affecting the *SLC16A2* gene at the X-breakpoint (17, 18). Furthermore, 2 other female patients with pathogenic variants and unfavorable X-chromosome inactivation similarly had a phenotype of equal severity to male patients with MCT8 deficiency (19, 20). However, a systematic clinical, biochemical, and molecular evaluation of females with features reminiscent of MCT8 deficiency is lacking. Also, potential correlations between the severity of the phenotype, the degree of X-chromosome inactivation, and residual TH transport function have not been studied so far.

Here, we document clinical, radiological, biochemical, and molecular features in 8 female patients with heterozygous pathogenic variants in *SLC16A2* and skewed X-chromosome inactivation, and explore correlations between the phenotypic spectrum and functional effect of genomic variations.

## Materials and Methods

### Patients

We studied 8 female patients with neurocognitive impairment and/or abnormal thyroid function tests in whom genetic analyses revealed a heterozygous pathogenic variant in *SLC16A2* in the context of regular care and for whom Erasmus Medical Center fulfilled a consultancy role. Furthermore, serum thyroid function tests of female family members of male patients—carrier and noncarrier—were retrieved from available historic data obtained in the context of clinical care (8).

### Ethical Considerations

Skin fibroblasts were collected for diagnostic purposes by the physicians in charge. Clinical examinations were carried out in the context of routine care and were retrospectively described. Where appropriate, this study was either ethically approved or the ethics committee provided a waiver for approval. Informed consent was obtained from the parents or legal representatives of all enrolled patients, unless the relevant institutional review board or local regulators had authorized the use of anonymized patient data without additional consent. This study was conducted in agreement with the Medical Research Involving Human Subjects Act.

### Clinical and Biochemical Evaluations

All patients were clinically evaluated by a trained (pediatric) neurologist and/or pediatric endocrinologist, and in a subset of cases a neuropsychologist. Imaging studies were performed in different centers using local scanning protocols. All biochemical measurements were carried out using standard laboratory methods.

### Patient Sequencing, Data Analysis, and Confirmation by Sanger Sequencing

Detailed methods on the (whole-exome or targeted) sequencing analyses and fluorescent in situ hybridization analyses are available in the supplementary material (**21**). Identified variants in *SLC16A2* were confirmed by Sanger sequencing on genomic DNA derived from leukocytes and patient-derived fibroblasts using primers that amplify the site of the variant for 4 patients (with missense or frameshift variants). Positions of the variants are determined using the NM_006517.3 reference sequence, which uses +1 as the A of the ATG translation initiation codon of the long MCT8 translational isoform, with the initiation codon as codon 1.

### X-chromosome Inactivation Studies

X-chromosome inactivation studies were carried out on DNA extracted from leukocytes and/or patient-derived fibroblasts using well-defined techniques used in routine clinical practice (HUMARA) (22).

### In Vitro Characterization of the Identified MCT8 Variants

Full technical details on the functional studies are available in the supplementary material (21). Briefly, COS-1 and JEG-3 cells were cultured and transfected as described previously (23). Two days after transfection, TH uptake studies, immunocytochemistry, and immunoblotting on total lysates and the surface biotinylated fraction were carried out using well-established protocols (23). See Supplementary Table S1 for the antibodies used in immunocytochemistry and immunoblotting (21). T3 uptake studies in patient-derived fibroblasts in the presence and absence of the MCT8-inhibitor silychristin were carried out as previously described (24).

### Statistical Analysis

All uptake results are expressed as means ± SEM of at least 3 independent experiments in duplicate. Statistical significance was determined using indicated statistical tests carried out in GraphPad Prism, version 9.

## Results

### Clinical and Biochemical Characterization of 8 Female Individuals With Neurocognitive Impairment and Abnormal Thyroid Function Tests

**Figure 1.**
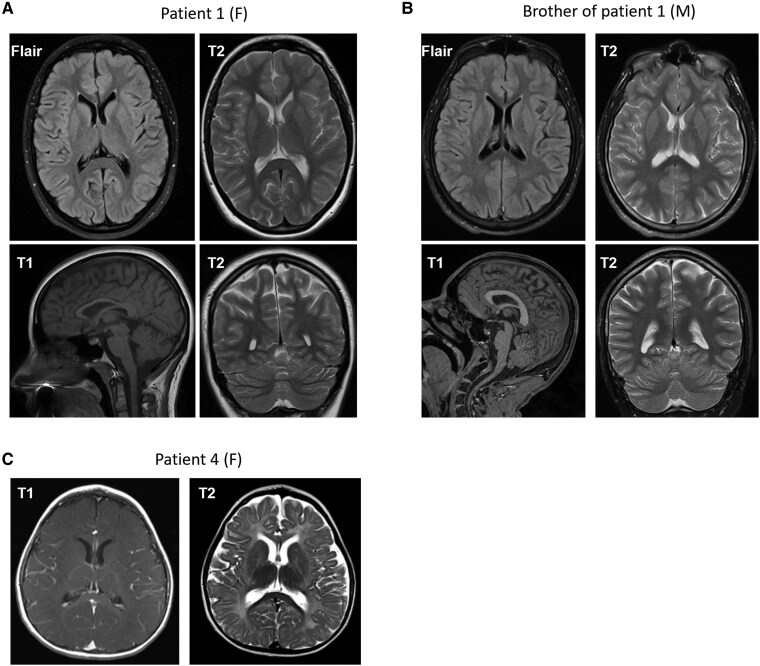
Magnetic resonance imaging (MRI) studies in patient P1 (female), her affected brother (patient P1B) and patient P4 (female). A, FLAIR-, T1-, and T2-weighted MRI of the brain of patient P1 performed at age 21 years, showing mild cortical atrophy without signs of leukodystrophy. B, FLAIR-, T1-, and T2-weighted MRI of the brain of the brother of patient P1 (patient IB) performed at age 25 years, showing signs of mild cortical atrophy without obvious signs of leukodystrophy. C, T1- and T2-weighted MRI of the brain of patient P4 at age 1 year show a small anterior cranial fossa and 6 to 7 month delayed myelination with nonmyelination in the superior and middle frontal gyrus. The applied MRI sequences are indicated in each image.

All female patients were evaluated for clinical outcomes associated with MCT8 deficiency in males (Table 1) (8). Three out of 8 female patients (P1, P3, and P8) showed mild-moderate intellectual disability (ie, IQ score <70), with speech development that was delayed or restricted to simple sentences and poorly developed social skills. In addition, all 3 patients exhibited behavioral problems including anxiety, depressive mood disorder, attention deficit disorder, or withdrawn behavior. Together, these features hampered participation in daily life activities and education. P2 and P6 had an IQ of 82 and 72, respectively. Brain magnetic resonance imaging (MRI) studies in patient P4 showed delayed myelination (Fig. 1C), in line with findings in male patients with MCT8 deficiency. In contrast to male patients with MCT8 deficiency, thyrotoxic symptoms were not prominent in the female patients. Detailed clinical and biochemical characterization of all female patients is provided in the supplementary material (21).

**Table 1.** Clinical and biochemical characterization of female patients with neurocognitive impairment and abnormal thyroid function tests

Patient	P1	P2	P3	P4	P5	P6	P7	P8
Sex	Female	Female	Female	Female	Female	Female	Female	Female
Age, y	25	5	15.5	2.5	5.9	2.3	2.3	10.3
Variant in *SLC16A2*	c.1333C > T p.(R445C)	c.578A > T p.(N193I)	c.826G > A p.(G276R)	t(X;20); breakpoint in intron 1	t(X;19); breakpoint in *SLC16A2*	c.1678delC p.(I562Sfs566*)	c.1333C > T p.(R445C)	c.661G > A p.(G221R)
Birth weight (SD)	−1.36	NA	NA	−0.34	1.09	−0.96	−0.60	0.04
**Endocrine features**								
Serum TT3, nmol/L	2.98 (1.4-2.5)	NA	2.55 (1.3-2.7)	4.06 (1.4-3.8)	NA	NA	NA	3.42 (1.8-3.0)
Serum FT3, pmol/L	7.70 (3.50-6.50)	7.20 (3.5-8.6)	NA	NA	8.10 (3.50-6.50)	7.57 (3.07-6.76)	5.99 (3.69-8.45)*^a^*	10.30 (5.10-7.40)
Serum TT4, nmol/L	74 (58-128)	NA	40 (70-150)	NA	NA	NA	NA	74 (63-124)
Serum FT4, pmol/L	12.0 (11.5-22.7)	11.6 (12.6-24.0)	8.0 (10.0-23.0)	10.6 (11.6-21.9)	8.2 (9.0-22.0)	9.8 (12.0-21.9)	23.2 (12.9-23.2)*^a^*	14.4 (13.1-24.0)
Serum rT3, nmol/L	0.19 (0.22-0.54)	NA	0.10 (0.11-0.40)	0.13 (0.15-0.37)	NA	NA	NA	0.13 (0.10-0.50)
Serum TSH, mU/L	2.22 (0.41-4.30)	4.30 (0.40-5.60)	2.10 (0.40-4.00)	0.17 (0.30-4.20)	7.70 (0.50-4.90)	2.49 (0.27-4.20)	2.38 (0.70-5.97)*^a^*	5.29 (0.60-5.20)
Serum SHBG, nmol/L	68 (20-120)	>180	NA	NA	NA	173 (60-190)	115 (40-140)*^a^*	115 (40-140)
Heart rate, bpm (percentile)	75 (50-75th)	70*^b^* (10-25th)	NA	110 (50th)	NA	120 (75-90th)	112 (50-75th)	75 (25th)
Body weight (SD), kg	80	25.9*^b^*	32.9 (0.20)*^c^*	11.5 (−1.10)	19.9 (−0.37)	14.0 (0.65)	12.0 (−0.50)	25.6 (−1.88)
Body height (SD), cm	161	127.5*^b^*	142.0 (−0.20)*^c^*	88.5 (−0.25)	111.5 (−1.34)	90.9 (0.38)	86.2 (−1.20)	129.4 (−2.46)
BMI (SD)	32.5	15.9	16.3 (0.20)*^c^*	14.6 (−1.25)	16.0 (0.61)	16.9 (0.68)	16.1 (0.12)	15.3 (−0.72)
Sweating	No	No	No	No	No	No	No	No
Diarrhea	No	No	No	No	Yes	No	No	No
**Neurological features**								
Dystonia	No	No	No	No	Yes	No	No	No
Spasticity	No	No	No	Yes	No	No	No	No
Hypotonia	No	No	No	Yes	Yes	No	No	No
Speech development	Simple sentences	Normal	Simple sentences	Cooing	Poor expressive language	Some words	Normal	Delayed
Head control	Yes	Yes	Yes	No	Yes	Yes	Yes	Yes
Sitting independently	Yes	Yes	Yes	No	Yes	Yes	Yes	Yes
Walking independently	Yes	Yes	Yes	No	Yes	Yes	Yes	Yes
Delay in achieving motor milestones	Yes	No	No	Yes	Yes	NA	No	Yes
GMFM-G88 score, %*^d^*	97.6	NA	NA	NA	NA	NA	NA	NA
VABS*^e^*—communication, percentile rank	<0.1	NA	NA	NA	NA	NA	NA	NA
VABS*^e^*—daily living skills, percentile rank	<0.1	NA	NA	NA	NA	NA	NA	NA
VABS*^e^*—socialization, percentile rank	<0.1	NA	NA	NA	NA	NA	NA	NA
IQ score (test)	45 (WAIS r)	82 (WISC-V)	53 (WISC-III)	NA	NA	72	NA	66 (SON-R)
Psychiatric symptoms	Depression, anxiety	No	Anxiety	No	Anxiety	No	No	Attention deficit disorder
EEG-proven seizures	No	No	No	No	No	No	No	No
Feeding problems	No	No	No	Yes	Yes	No	No	No
Brain MRI abnormalities	Yes	No	No	Yes	Yes	No	NA	No

Abbreviations: BMI, body mass index; bpm, beats per minute; EEG, electroencephalogram; FT3, free 3,5,3′-triiodothyronine; FT4, free thyroxine; MRI, magnetic resonance imaging; NA, not available; rT3, 3,3,5′-triiodothyronine; SHBG, sex hormone–binding globulin; TSH, thyrotropin; TT3, total 3,5,3′-triiodothyronine; TT4, total thyroxine.

^*a*^Thyroid function tests were evaluated under levothyroxine supplementation (60 µg; 5 µg/kg/day).

^*b*^The latest available body weight, height, and heart rate are provided, measured at age 7 years.

^*c*^The latest available body weight, height, and BMI are provided, measured at age 9 years and 10 months.

^*d*^GMFM-G88 stands for Gross Motor Function Measure-G88. The GMFM-G88 assesses the gross motor function in which scores range from 0% to 100%, with higher scores indicating better motor function and where a 100% score is achieved by a normal developing child of age 4 years.

^*e*^VABS stands for Vineland Adaptive Behavior II. The VABs II is a survey that assesses different aspects of development. The survey was completed by primary caregivers in presence of a trained neuropsychologist or physician. The level of development on indicated subdomains of the VABs II is provided as percentile ranks and reflects the percentage of individuals in the patients’ normative age group who scored the same or lower than the patient.

### Molecular Genetic Studies

Since routine diagnostics did not identify the underlying cause of the observed phenotype in female patients P1, P3 to P5, and P7 and P8, exome sequencing was carried out. Sanger sequencing was performed for P6 as she had a brother with a classical phenotype of MCT8 deficiency. In P2, targeted sequencing was performed. A heterozygous variant NM_006517.3(*SLC16A2*):c.1333C > T; p.(R445C) was identified in patient P1, which has been previously reported in a male patient with MCT8 deficiency (25). Skewing analyses in leukocytes indicated nonrandom X-inactivation (90:10). The same genetic variant was identified in her older brother (patient 1B), but not in other family members. In patient P2 a heterozygous c.578A > T, p.(N193I) variant was found. X-inactivation in leukocytes was skewed (∼90:10). In patient P3 a heterozygous de novo variant c.826G > A, p.(G276R) in *SLC16A2* was identified, known to result in severe MCT8 deficiency in male patients (26). Analyses of the X-chromosome inactivation pattern in leukocytes showed a 60:40 skewing ratio. However, skewing was more pronounced in patient-derived fibroblasts (85:15). In patient P4, karyotyping revealed a de novo reciprocal translocation between the long arm of chromosome X at band Xq12 and the long arm of chromosome 20 at band 20q11.2 (46,X,t(X;20)(q12;q11.2)). A chromosome microarray analysis did not detect any copy number variations, suggesting that this translocation was balanced. To better characterize this patient's translocation and to obtain higher resolution details about its breakpoint, Mate-Pair Whole Genome Sequencing was performed, which revealed that the X;20 translocation resulted in disruption of *SLC16A2*, with the breakpoint located within intron 1 of the *SLC16A2* gene (seq[GRCh38] t(X;20)(Xpter->Xq13.2::20q13.11->20qter;20pter->20q13.11::Xq13.2->Xqter)). This results in functional inactivation of *SLC16A2*. X-inactivation studies were not performed. In patient P5, a balanced translocation between the X chromosome and chromosome 19 was observed on karyotype. Subsequent fluorescent in situ hybridization analyses pinpointed the breakpoint to the *SLC16A2* gene, as a probe aligned to a substantial part of *SLC16A2* resulted in fluorescence on the intact X-chromosome and both the derivative X-chromosome and the derivative chromosome 19 (Supplementary Table S2) (21), disrupting its function. In leukocytes of P5, skewed X-inactivation at a ratio of 80:20 was observed. Targeted sequencing of *SLC16A2* was performed in P6, as her brother had been previously diagnosed with MCT8 deficiency, and yielded a single-nucleotide deletion resulting in a frameshift (c.1678delC, p.(I562Sfs566*)). This variant was considered pathogenic due to its truncating nature before the C-terminal tail (27). X-chromosome inactivation analyses demonstrated prominent skewing both in leukocytes (90:10) and patient-derived fibroblasts (98:2). In patient P7, a heterozygous de novo c.1333C > T, p.(R445C) variant was found in the *SLC16A2* gene through next-generation sequencing panel sequencing, which was identical to the variant identified in female patient P1. The X-inactivation analysis of this patient's leukocytes showed a skewing ratio of 58:42, in patient-derived fibroblasts this ratio was 81:19. Female patient P8 showed a de novo heterozygous missense variant (c.661G > A, p.(G221R)) in the *SLC16A2* gene. Analysis of X-inactivation in leukocytes showed a skewing ratio of 54:46, while in patient-derived fibroblasts this ratio was 93:7. In all female patients for whom exome sequencing was carried out (P1-P5, P7 and P8), no other disease-causing variants were discovered.

### Functional Studies

**Figure 2.**
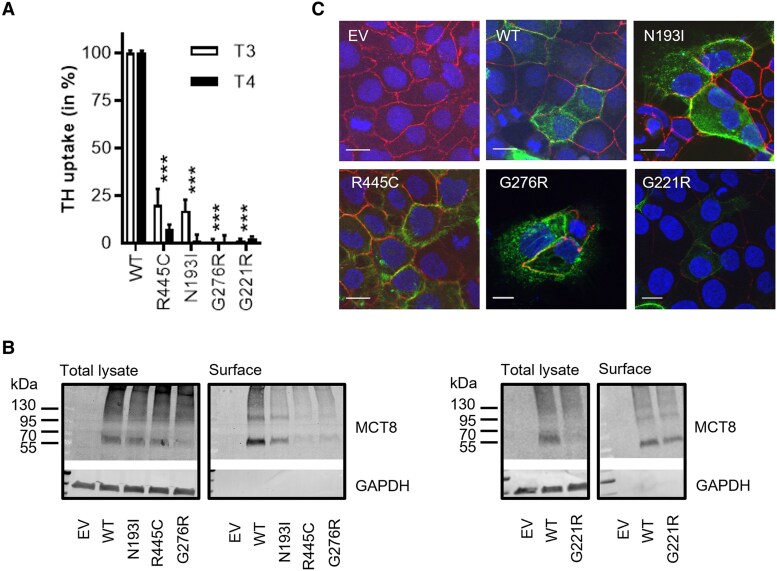
In vitro functional studies confirmed pathogenicity of identified mutations. A, T3 and T4 uptake studies in COS-1 cells transiently transfected with wild-type (WT) MCT8 or indicated mutants and the intracellular thyroid hormone (TH)-binding protein CRYM in 30 minutes at 37 °C. Data are expressed relative to WT MCT8 and are corrected for background TH uptake in empty vector transfected control cells. Results are presented as means ± SEM (N = 3-4). One-way analysis of variance with Dunnett post hoc tests were used to assess for statistically significant differences compared to healthy controls (*P < .05; **P < .005; ***P < .0005; ****P < .0001). B, Representative immunoblot analyses of MCT8 protein in total lysates (left) and the surface biotinylated fraction (right) of COS-1 cells transfected with indicated constructs. GAPDH (glyceraldehyde-3-phosphate dehydrogenase) was used as a loading control. Immunoblot analyses of G221R have been performed separately. C, Immunocytochemistry in transfected JEG-3 cells to visualize WT and mutant MCT8 (green) subcellular localization. Zona occludens (ZO-1; red) was used as a membrane marker and DAPI (4′,6-diamidino-2-phenylindole; blue) as a nuclear marker.

To substantiate the diagnosis of MCT8 deficiency in the female patients, we evaluated the effect of the identified missense variants in vitro. On transient transfection in COS-1 cells, both T3 and T4 uptake by the N193I, G276R, G221R, and R445C mutant MCT8 proteins were strongly diminished compared to wild-type (WT) (Fig. 2A). Similar results were obtained in JEG-3 cells (Supplementary Fig. S1) (21). Although all mutants could be detected in the cell surface fraction, the total and cell surface expression levels of 3 out of 4 mutants (R445C, G276R and G221R) were considerably lower than WT (Fig. 2B). Accordingly, immunocytochemistry in JEG-3 cells showed the presence of all 4 mutants at the cell membrane (Fig. 2C). The effect of the other variants was not evaluated in vitro, either because this was technically impossible (chromosomal translocations) or because of their deleterious nature (frameshift).

**Figure 3.**
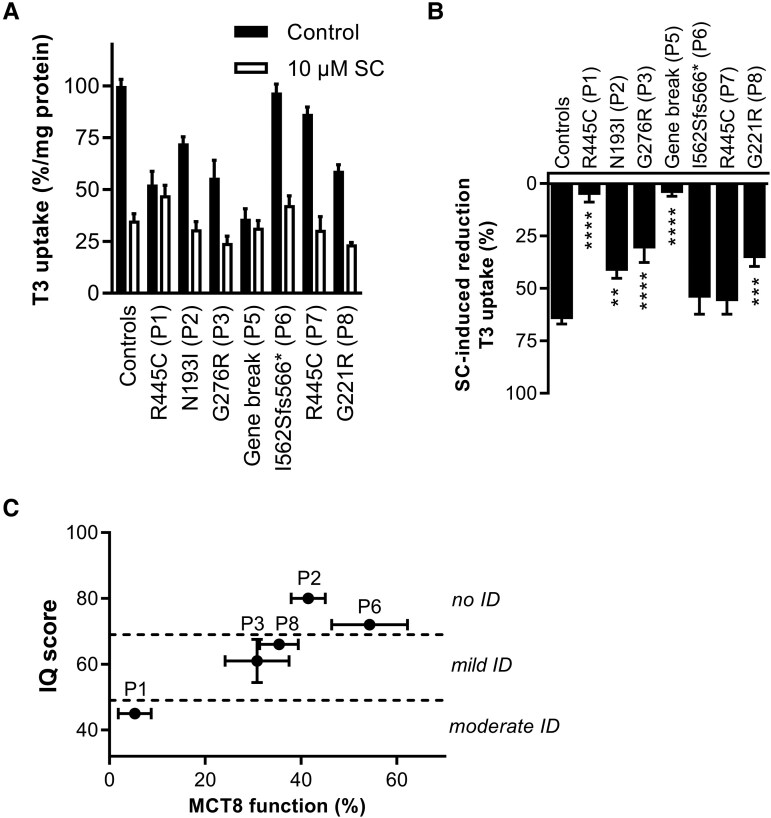
Ex vivo analyses of MCT8 function in patient-derived fibroblasts. A, Triiodothyronine (T3) uptake in patient and control fibroblasts in the absence (black) and presence (white) of 10-µm silychristin (SC), a specific inhibitor of MCT8. Data are expressed relative to the amount of T3 accumulation in control cells in the absence of SC. B, Silychristin-induced reduction in T3 uptake in control and patient fibroblasts. Data are derived from A. One-way analysis of variance with Dunnett post hoc tests were used to assess for statistically significant differences compared to healthy controls (*P < .05; **P < .005; ***P < .0005; ****P < .0001). C, Correlation plot of patient’s IQ (divided into no, mild, and moderate intellectual disability (ID)) vs MCT8 function, defined as percentage silychristin-induced reduction in T3 uptake shown in B. In case of multiple available measurements, the mean ± SEM are displayed.

Next, we studied T3 uptake in fibroblasts derived from 7 female patients (P1-P3 and P5-P8) and healthy controls. T3 uptake levels in the fibroblasts derived from the female patients P1 to P3, P5, and P8 were significantly lower than in control fibroblasts. The T3 uptake capacity in the R445C mutant fibroblasts derived from one female patient (P1) was similar to male MCT8-deficient patients. In contrast, T3 uptake levels in the R445C mutant fibroblasts derived from female patient P7 were more similar to those in control fibroblasts, which was also the case for female patient P6 (Fig. 3A). Similar to control fibroblasts, a time-dependent increase of T3 uptake was observed in all the fibroblasts of female patients (P1-P3), compatible with the relatively mild clinical phenotype of these patients compared to affected males (Supplementary Fig. S2A) (21). In line with the in vitro overexpression studies, the expression levels of the G276R (P3) and R445C (P1) mutants were strongly reduced compared to WT, while expression levels of N193I (P2) were similar to WT (Supplementary Fig. S2B) (21). To confirm that the impaired T3 transport was due to a reduced transport capacity of MCT8, we performed T3 uptake studies in the presence and absence of the MCT8-specific inhibitor silychristin (28). Indeed, T3 uptake was less reduced by silychristin in the patient-derived fibroblasts of P1 to P3, P5, and P8 than in controls, strongly supporting MCT8 dysfunction in cells derived from female patients, but not in fibroblasts derived from P6 and P7 (Fig. 3B). For female patients, the silychristin-induced reduction of T3 uptake in fibroblasts (reflecting residual MCT8 transport capacity) showed a positive trend with available IQ scores (Fig. 3C).

### Comparison of Female Index Patients to Asymptomatic Carriers and Noncarriers

**Figure 4.**
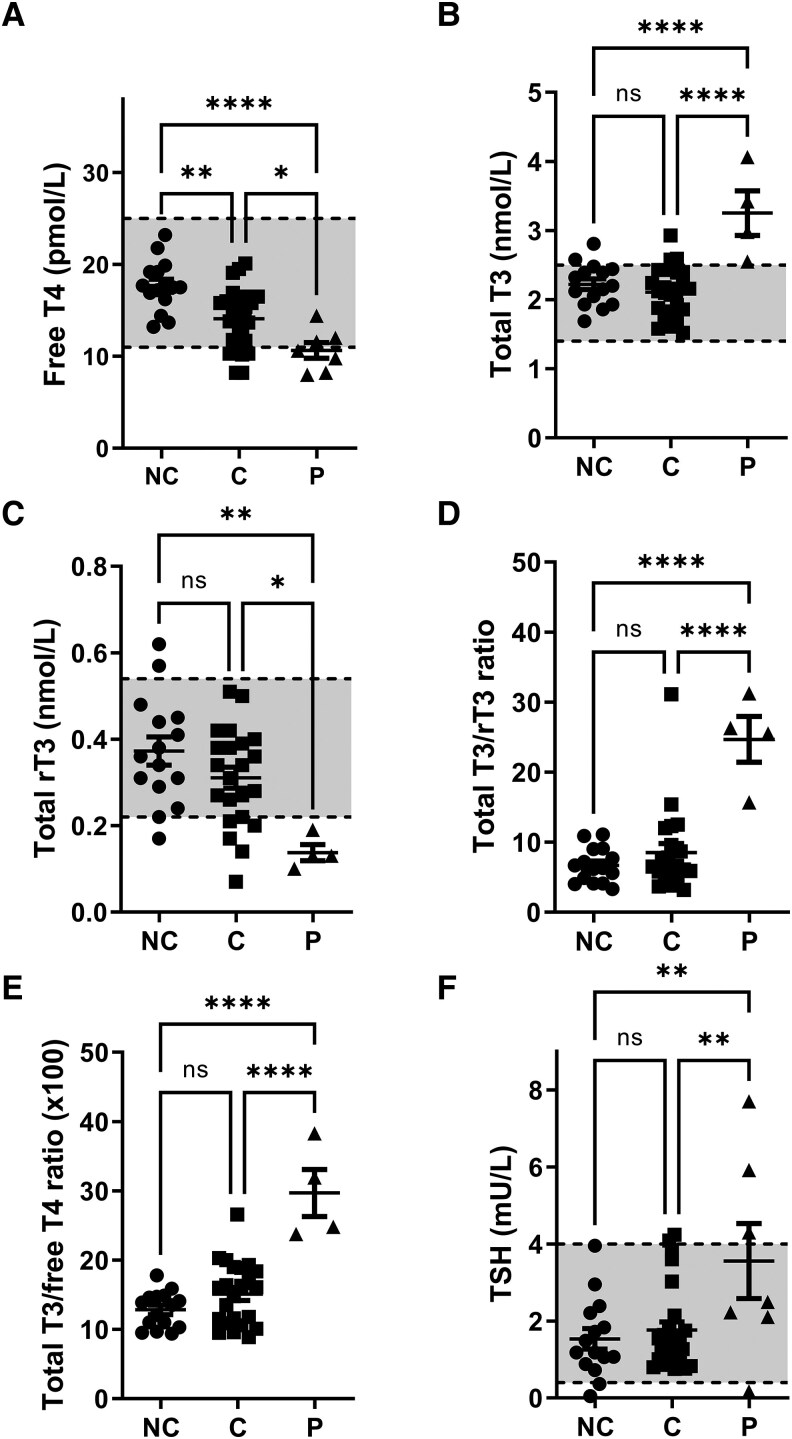
Serum thyroid function tests in female patients in comparison to asymptomatic carriers and noncarriers. A, Serum free thyroxine (T4); B, total triiodothyronine (T3); C, total 3,3,5′-triiodothyronine (rT3) concentrations; D, total T3/total rT3; and E, total T3/free T4 ratios; and F, thyrotropin (TSH) concentrations in female patients (*P*), asymptomatic female carriers (C) and noncarriers (NC). One-way analysis of variance with Tukey post hoc tests were used to test for statistically significant differences between groups (ns, not significant; *P < .05; **P < .005; ***P < .0005; ****P < .0001).

To further substantiate that the observed clinical features are related to the identified variants in *SLC16A2*, we compared the serum thyroid function tests of the female index patients to those available from (self-reported) asymptomatic carriers and noncarriers. Patient P7 was excluded from these analyses, as thyroid functions tests of this index patient were available only on levothyroxine supplementation. The other female patients (P1-P6 and P8) showed significantly lower serum free T4 concentrations than asymptomatic carriers and noncarriers (Fig. 4A). In addition, serum total T3 concentrations significantly exceeded those observed in asymptomatic carriers (Fig. 4B). Total reverse T3 (rT3) concentrations in the female patients were significantly lower compared to noncarriers and were similar to the lowest quartile of asymptomatic carriers (Fig. 4C). This resulted in a pronounced increase in the total T3/total rT3 and total T3/free T4 ratios in the female patients (Fig. 4D and 4E). Although TSH concentrations were more variable, they were significantly higher in the patient group (Fig. 4F). Together, the thyroid function tests in the female patients clearly differed from those observed in asymptomatic carriers and noncarriers, resembling the biochemical signature of MCT8 deficiency reported in affected males, although to a lesser extent.

## Discussion

Here, we report on the clinical, radiological, biochemical, and molecular phenotypes associated with heterozygous pathogenic variants in the *SLC16A2* gene and skewing of X-chromosome inactivation in 8 female patients. The major presenting features were variable neuropsychological abnormalities, behavioral features, and abnormal serum thyroid function tests. The causality of these *SLC16A2* variants to the phenotype was supported by functional studies in patient-derived fibroblasts that showed a reduction in MCT8-mediated T3 uptake in 5 out of 8 female patients, with a similar magnitude as observed in fibroblasts from affected males.

Two female patient cases (P4 and P5) exemplify how pathogenic variants in *SLC16A2* may lead to manifestations of equal severity as in males with MCT8 deficiency, as has been reported before (17-19). Disease features were considerably milder in the other female patients (P1-P3 and P6-P8). Especially motor function and development were less affected, and key neurological features typically present in affected males, such as hypotonia, dystonia, and spasticity, were either absent or present at a very mild degree. Four of such female individuals presented with withdrawn behavior and psychiatric symptoms, such as anxiety, depression, or an attention deficit disorder that have not been previously described in males. We postulate that the homogeneous inactivation of MCT8 in male individuals and female individuals with almost complete skewing (eg, due to balanced X-autosomal translocations generally leading to inactivation of the “normal” X-chromosome (29), such as in P4 and P5) may lead to severe global developmental delay, concealing the effect of MCT8 inactivation on more complex brain functions such as behavior and emotion regulation. In female individuals with pathogenic variants that allow a heterogeneous (cell type–specific) skewing profile (ie, P1-P3 and P6-P8), the effect of suboptimal MCT8 function on emotion regulation and higher cognitive functions may become clinically evident. We speculate that these female patient cases may also illustrate clinical presentations associated with variants resulting in mild loss-of MCT8 function in male individuals, who may have remained undiagnosed thus far.

The TH profile of all female index patients clearly differed from asymptomatic carriers and noncarriers with the total T3/total rT3 and total T3/free T4 ratios being elevated, confirming previous findings suggesting that elevated free T3/free T4 ratios can be used in (female) patients with developmental delay to indicate MCT8 deficiency or other TH resistance syndromes (30). This signature is reminiscent of the abnormal thyroid function tests observed in males with MCT8 deficiency, but less pronounced (31). This potentially explains the absence of evident thyrotoxicity in peripheral tissues in the affected female patients. In the absence of clear thyrotoxic features, treatment with TH analogues should be considered on a case-to-case basis.

The strong effects observed in our functional studies contrast with the milder motor and neurocognitive impairment in 4 out of 8 female patients (P1-P3 and P8). This suggests that at least a proportion of cells in the blood-brain barrier and neural cells express the WT allele, which can substantially modify the phenotype. Although X-chromosome inactivation in humans is random and initiated during early development (∼day 5.5 after fertilization), resulting in a relatively homogenous profile across tissues, variability does exist (32-34). This variability may explain the absence of skewing in the leukocytes of patient P3, while there was pronounced skewing and clearly impaired MCT8 function in patient-derived fibroblasts. Such variability could also explain the discordance between abnormal thyroid function tests and otherwise normal development as well as the normal T3 uptake in cells with a presumed skewing to the WT allele such as in P6 and P7. In addition to distinct skewing profiles across cell types, some genes reportedly escape X-chromosome inactivation when in close (3-dimensional) proximity to the X-inactivation regulator long noncoding RNA X-inactive specific transcript (*XIST*), such as *SLC16A2* (32, 35, 36). This escape phenomenon may be active in our fibroblast disease model (37). Moreover, natural selection of fibroblasts expressing the unaffected X-chromosome may have occurred during culture, resulting in considerable T3 uptake capacity in some female cases (P6 and P7). Taken together, it is conceivable that some tissues or even cell populations in our female patients express the WT MCT8 allele, and hence ensure adequate intracellular TH availability, which importantly determines disease penetrance.

Our study has several limitations. First, although we believe a causal relation between genotype and phenotype is most plausible in the presence of a pathogenic variant in *SLC16A2* with skewed X-chromosome inactivation, characteristic thyroid function tests, and defective MCT8-mediated T3 transport in patient cells, demonstrating causality can be challenging in case information regarding some of these diagnostic hallmarks is lacking (ie, in P7). Second, due to the unavailability of detailed clinical information and patient-derived fibroblasts of presumed asymptomatic female carriers in whom thyroid function tests were performed, we were unable to prove causality between genotype and phenotype. Nevertheless, most asymptomatic female carriers with no evidence for skewed X-inactivation lack neurocognitive impairment or endocrine abnormalities (13). Although low-normal serum (free) T4 and relatively high serum T3 concentrations have been reported in some asymptomatic female carriers, the implications of such isolated endocrine abnormalities are currently unknown (7, 14). Overall, it is conceivable that these clinical and biochemical features arise from some degree of skewed X-chromosome inactivation. Despite the aforementioned limitations, our studies suggest that ex vivo studies in fibroblasts may be a suitable strategy to support our hypothesis.

In conclusion, the present work demonstrates how heterozygous variants of *SLC16A2* in female patients with skewed X-chromosome inactivation display similar biochemical features and variably the neurocognitive and radiological abnormalities seen in males with MCT8 deficiency. Moreover, a spectrum of psychiatric disease features is seen in affected females that has not been previously described in males. Our findings indicate that MCT8 deficiency should be considered in females presenting with mild-moderate neurocognitive impairment with or without psychiatric symptoms and abnormal thyroid function tests (low T4, high T3). Further research is needed to more fully define the potential impairment in female carriers as well as the potential therapeutic benefit of TH analogues.

## Data Availability

All data sets generated and/or analyzed during the present study are not publicly available to preserve patient confidentiality, but are available from the corresponding author on reasonable request.

## Abbreviations

BMI: body mass index
MCT8: monocarboxylate transporter 8
MRI: magnetic resonance imaging
rT3: 3,3,5′-triiodothyronine
T3: 3,5,3′-triiodothyronine
T4: thyroxine
TH: thyroid hormone
TSH: thyrotropin (thyroid-stimulating hormone)
WT: wild-type
